# Supplementary material for: Molecular assembly of the period-cryptochrome circadian transcriptional repressor complex
Source: eLife. 2014 Aug 15;3:e03674. doi: 10.7554/eLife.03674 (PMC4157330; doi:10.7554/eLife.03674)
Supplement: Figure 1—source data 1. — Each individual mutation was unable to disrupt PER-CBD-CRY1 binding. Only the combined mutation of two nearby stretches of residues on the C-terminal half of PER2-CBD was able to abolish binding. DOI: http://dx.doi.org/10.7554/eLife.03674.004 [file elife03674s001.pdf]

Construct name	Insert information	Vector	AA	MUT
pCIn-Per2NF	Per2 with NLS and FlagTag at C-terminus	pCIneo SalI/NotI		
pCIn-Per2mIK	Per2NF carrying mutation in CBD	pCIneo NheI/NotI	IK	AA
pCIn-Per2mVLQDP	Per2NF carrying mutation in CBD	pCIneo NheI/NotI	VLQDP	AAAAA
pCIn-Per2mIW	Per2NF carrying mutation in CBD	pCIneo NheI/NotI	IW	AA
pCIn-Per2mLLM	Per2NF carrying mutation in CBD	pCIneo NheI/NotI	LLM	AAA
pCIn-Per2mMMTYQ	Per2NF carrying mutation in CBD	pCIneo NheI/NotI	MMTYQ	AAAAA
pCIn-Per2mPSRD	Per2NF carrying mutation in CBD	pCIneo NheI/NotI	PSRD	AAAA
pCIn-Per2mVLK	Per2NF carrying mutation in CBD	pCIneo NheI/NotI	VLK	AAA
pCIn-Per2mEKL	Per2NF carrying mutation in CBD	pCIneo NheI/NotI	EKL	AAA
pCIn-Per2mQPRF	Per2NF carrying mutation in CBD	pCIneo NheI/NotI	QPRF	AAAA
pCIn-Per2mQRREL	Per2NF carrying mutation in CBD	pCIneo NheI/NotI	QRREL	AAAAA
pCIn-Per2NFmutA	Per2NF carrying mutation in CBD	pCIneo NheI/NotI	see below	see below
pCIn-Per2NFmutB	Per2NF carrying mutation in CBD	pCIneo NheI/NotI	see below	see below

```
      1130                                                        1190
PER2  IKYVLQDPIWLLMANTDDSIMMTYQLPSRDLQAVLKEDQEKLKLLQRSQPRFTEGQRRELR
mutA  ----AAAAA---------------------------------AAAA---------------------------------------------------------------
mutB  -------------------------------------------------------------------------------------------------AAAA-----AAAAA--
```

Figure 1—source data 1
